# Cross-Sectoral Comparisons of Process Quality Indicators of Health Care Across Residential Regions Using Restricted Mean Survival Time

**DOI:** 10.1097/MLR.0000000000002057

**Published:** 2024-10-11

**Authors:** Hana Šinkovec, Walter Gall, Georg Heinze

**Affiliations:** * Institute of Clinical Biometrics, Center for Medical Data Science, Medical University of Vienna, Vienna, Austria; † Biotechnical Faculty, University of Ljubljana, Ljubljana, Slovenia; ‡ Institute of Medical Information Management, Center for Medical Data Science, Medical University of Vienna, Vienna, Austria

**Keywords:** health care provider comparisons, pseudo-observations, risk adjustment, restricted mean survival time, restricted mean time lost

## Abstract

**Background:**

Practice guidelines recommend patient management based on scientific evidence. Quality indicators gauge adherence to such recommendations and assess health care quality. They are usually defined as adverse event rates, which may not fully capture guideline adherence over time.

**Methods:**

For assessing process indicators where compliance to the recommended treatment can be assessed by evaluating a patient's trace in linked routine databases, we propose using restricted mean survival time or restricted mean time lost, which are applicable even in competing risk situations. We demonstrate their application by assessing the compliance of patients with acute myocardial infarction (AMI) to high-power statins over 12 months in Austria’s political districts, using pseudo-observations and employing causal inference methods to achieve regional comparability.

**Results:**

We analyzed the compliance of 31,678 AMI patients from Austria’s 116 political districts with index AMI between 2011 and 2015. The results revealed considerable compliance variations across districts but also plausible spatial similarities.

**Conclusions:**

Restricted mean survival time and restricted mean time lost provide interpretable estimates of patients’ expected time in compliance (lost), well-suited for risk-adjusted entity comparisons in the presence of (measurable) confounding, censoring, and competing risks.

Practice guidelines distill scientific evidence and guide health care providers in recommending management strategies for patients, potentially leading to optimal outcomes and cost reduction.^1^ Quantifying adherence to these guidelines (eg, by profiling hospital performance) serves as a direct measure of care quality and a basis for improvement.^2^ This is crucial for different stakeholders, hospitals, doctors in private practice, and patients. Routinely collected health care claims provide a rich data source for such evaluations, supplying real-world evidence on care delivery, nationwide adherence estimates, identification of nonadherence risk factors, regional and provider outcome comparisons, and trend tracking. However, employing health care claims for quality assessment across different entities [where an entity could denote a provider (eg, hospital) or a residential region of patients] represents an important statistical challenge.

First, to remove individual confounding biases and obtain comparable estimates of compliance to treatment guidelines across entities, risk adjustment is crucial.^3,4^ To adjust for different case mixes of patients across entities, causal inference methods should be adopted wherein the focus is on estimating for each entity, the average of the potential outcomes of all patients of the population, had they all been assigned to that particular entity. This involves identifying potential confounders and their functional forms to avoid model misspecification.^5,6^ Including a comprehensive set of confounders is likely to enhance result accuracy and interpretation, as the optimal adjustment for patient characteristics in practice remains uncertain.^7^ However, when the data lack adequate patient numbers per entity, further problems known as sparse data bias may arise.^6–8^ To avoid potential model misspecifications, doubly robust estimators combining both outcome modelling and exposure modelling are appealing because unbiased estimates are obtained when at least one of the 2 models is correctly specified, giving the researcher “two opportunities to get it right.”^7,9^

Second, in the literature, provider performance is often assessed using binary quality indicators, yielding simple surrogate measures of care quality with clear interpretation, such as the proportion of correctly treated patients.^10^ For example, indication indicators check if the diagnosis matches therapy, outcome indicators estimate outcome rates (eg, mortality, complication, readmission), and process indicators (PI) evaluate adherence of processes to medical guidelines. While many countries use such indicators for inpatient care, guideline-compliant treatment during outpatient follow-up, like prophylactic medication, is relevant for patients in the long term.^11^ For instance, patients with acute myocardial infarction (AMI) should receive lifelong high-intensity statin therapy after discharge. PI can be assessed by evaluating the traces of patients in linked routine databases, typically available to social insurance institutions. However, a pure binary evaluation after a certain time (measuring full adherence to practice guidelines until some relevant time point) may miss the variation in the extent of partial compliances. Moreover, patients may not be observable for the full study period after their index event because of competing events or loss to follow-up.

In this paper, we propose restricted mean survival time (RMST) or restricted mean time lost (RMTL) as alternative estimands for evaluating PI.^12^ Methods section and Supplement (Supplemental Digital Content 1, http://links.lww.com/MLR/C883)​​​​​ detail their risk-adjusted estimation, including assumptions, estimation, extensions, and interpretation. We apply these methods to compare patients’ expected time in compliance (lost) with high-intensity statin therapy over 12 months post-AMI across Austria’s political districts, presented in Results section. The paper closes with a discussion of our main findings.

## METHODS

### Motivating Example

Assume that *Y* is a random variable denoting the time to the occurrence of an event of interest. Let *C* be the censoring time, and δ=I(Y≤C) an indicator of noncensoring. Due to right censoring, the mean value of Y is not easy to estimate nonparametrically. Instead, the RMST is the expected survival time restricted to a maximum time τ. ^12^ In the absence of covariates, it is defined as the area under the survival curve over the time interval [0, τ],


$$E\left(Y\wedge \tau \right)=\int_{0}^{\tau }S\left(t\right)\mathrm{dt},$$

where $S\left(t\right)=P(Y>t)$ is the all-cause survival function over time t . In the analysis of PI, we consider *Y* as the time to the first nonadherence to treatment guidelines (cessation of therapy). The estimates provided by the RMST can be interpreted as the expected time in compliance before time τ or alternatively, we can consider RMST /τ as the fraction of the total time in compliance. The truncation time point τ can be chosen as either the recommended or clinically relevant duration of therapy (eg, for lifelong treatments). The risk-adjusted RMST for each entity Z (eg, regions, hospitals, patients’ residential regions) ( Z=1,…,m ) can then be compared across entities to assess variations in adherence to PI. Analogously, the restricted mean time lost (RMTL) is given by:


$$\mu =\tau -E\left(Y\wedge \tau \right)=\tau -\int_{0}^{\tau }S\left(t\right)\mathrm{dt},$$

and could be interpreted as the expected time in compliance lost before time τ .

**FIGURE 1:**
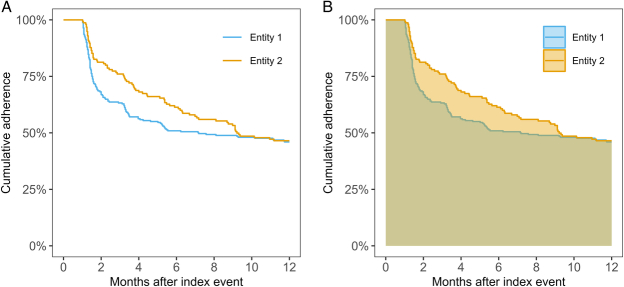
(A) Kaplan-Meier estimates of cumulative adherence in 2 entities and (B) area under the survival curve representing mean time in compliance restricted to 12 months in the 2 entities.

To motivate the RMST estimand for assessing PI (eg, statin use in post-AMI patients), consider the unadjusted cumulative adherence to treatment guidelines of patients from 2 different entities using the Kaplan-Meier method, shown in Figure 1A. These patients were followed up over a recommended treatment period of τ= 12 months. In a binary evaluation, one would restrict the analysis to patients that were fully observed until time τ and then compute the rates of compliant patients in the 2 regions. However, a pure binary evaluation (patient fully adhered to practice guidelines until time τ or not) may miss the temporal variation in the extent of partial compliances. Moreover, patients may not be observable for the full period after their index event because of loss to follow-up or administrative censoring. Alternatively, the intensity of noncompliance per τ= 12 months could be computed, where the number of patients who became noncompliant during follow-up is related to the total number of follow-up times, restricted to τ=12 months per patient. The intensity of compliance is then given by the complement of the intensity of noncompliance. Lastly, cumulative probabilities of compliance at time τ could be evaluated (Table 1).

**TABLE 1:** Four Estimands to Define Compliance, Corresponding Estimators, and Estimates for 2 Entities, as Shown in Figure 1

Estimand	Estimator	Estimate of compliance for entity 1 (95% CI)	Estimate of compliance for entity 2 (95% CI)
Probability of compliance when fully observed up to 12 months	Relative frequency (binomial)	0.48 (0.41, 0.54)	0.47 (0.39, 0.55)
Intensity of compliance at 12 months	Maximum likelihood estimator of rate parameter (Poisson)	0.46 (0.39, 0.53)	0.47 (0.39, 0.55)
Cumulative probability of compliance up to 12 months	Kaplan-Meier	0.46 (0.40, 0.53)	0.47 (0.39, 0.55)
Expected proportion of time in compliance within 12 months	RMST	0.58 (0.53, 0.63)	0.65 (0.59, 0.71)

RMST indicates restricted mean survival time.

Unlike these approaches (or alternative methods for time-to-event analysis), the RMST (or RMTL) can summarize the expected time in compliance (lost) and may indicate differences also when noncompliance initially diverges and later converges (Figure 1B). It places more emphasis on compliance in the initial months, which is probably more relevant for a patient than compliance close to time τ (Table 1). This estimand is free of model assumptions and has a natural causal interpretation.^13^

### Competing Risks

In situations where other, j=1,…,J , competing events may prevent the event of interest from occurring, and D denotes the cause of failure, the cause-specific cumulative incidence function, describing the probability of j event occurring before time t while also being at risk of other competing events, is defined as:


$$F_{j}\left(t\right)=P\left(Y\leq t,\;D=j\right).$$

As Andersen^14^ showed, the (total) RMTL μ before time τ could be decomposed into a sum over failure causes j of terms:


$$S\left(t\right)=1-\sum_{j=1}^{J}F_{j}\left(t\right).$$

Thus, the RMTL can be written as a function of each cause-specific cumulative incidence function as follows:


$$E\left(Y\wedge \tau \right)=\int_{0}^{\tau }S\left(t\right)\mathrm{dt}=\int_{0}^{\tau }1-\sum_{j=1}^{J}F_{j}\left(t\right)\mathrm{dt}=\tau -\int_{0}^{\tau }\sum_{j=1}^{J}F_{j}\left(t\right)\mathrm{dt}.$$

Reformulating the equation above as:

$$\mu =\tau -E\left(Y\wedge \tau \right)=\tau -\int_{0}^{\tau }S\left(t\right)\mathrm{dt}=\sum_{j=1}^{J}\int_{0}^{\tau }F_{j}\left(t\right)\mathrm{dt},$$

to obtain the RMTL that can be further partitioned into:


$$\mu_{j}=\int_{0}^{\tau }F_{j}\left(t\right)\mathrm{dt},$$

that is, the area under cumulative incidence function $F_{j}\left(t\right)=P\left(Y\leq t,D=j\right)$ over [0,τ] , yields an estimand of the RMTL due to event type j before time τ. ^14–16^

**FIGURE 2:**
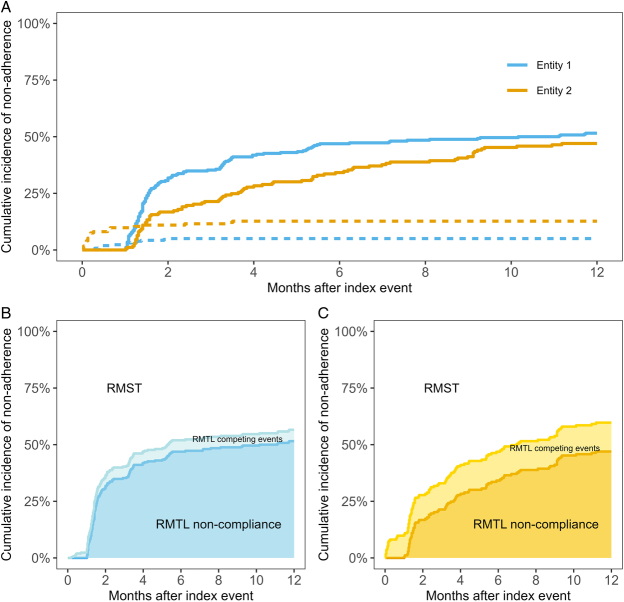
(A) Cumulative incidence of nonadherence (solid lines) and competing events (dashed lines) in 2 entities. Representation of mean time in compliance (RMST), mean time lost due to nonadherence (RMTL noncompliance), and mean time lost due to competing events such as death and reinfarction (RMTL competing events) in entity 1 (B) and entity 2 (C). RMST indicates restricted mean survival time; RMTL, restricted mean time lost.

Graphically, RMST and RMTL before τ , j=1,…,J , can be illustrated as in Figures 2B and C, expanding on the previous section example to the competing risks situation where the time to nonadherence to treatment guidelines ( j=1 ) may also be subject to death or recurrent AMI ( j=2 ) (Fig. 2A). The cumulative incidences may be presented on a “stacked” plot, where $\mu_{j=1}$ before τ is the area under the lower curve between 0 and τ and $\mu_{j=2}$ before τ is the area between the 2 curves between 0 and τ . Finally, $E\left(Y\wedge \tau \right)$ is the area between the upper curve and 1 between 0 and τ. ^14^

RMST and RMTL can be statistically modelled using pseudo-observations,^17^ see Supplemental Section S1 (Supplemental Digital Content 1, http://links.lww.com/MLR/C883). Risk adjustment, that is, comparison of standardized RMST between entities, relies on methods that build on assumptions for causal inference, which are outlined in Supplemental Section S2 (Supplemental Digital Content 1, http://links.lww.com/MLR/C883).

In the following, we employ the methodology outlined above to evaluate the utilization of high-intensity statins, which are recommended for all patients with AMI regardless of cholesterol concentration and age,^18,19^ over a 12-month period after AMI at the regional level, using Austrian claims data.

### Data Sources

The data provided by the Main Association of Austrian Social Insurance Institutions encompassed inpatient and outpatient medical services covered by individual health insurance funds. These funds are determined based on patients’ employment or province of residence, excluding private insurance. With health insurance being mandatory in Austria, this dataset represents over 98% of the Austrian population. The data included patients’ demographic information with date of death (if applicable) and claims filled between 2011 and 2015. In particular, it contained information on all drug prescriptions (number of packages, package size, and dose per unit) as well as hospital data with primary and associated diagnoses at hospital discharge. Prescriptions were encoded with a unique Austrian pharmaceutical registration number linked to the Anatomical Therapeutic Chemical (ATC) Classification System,^20^ and hospital discharge diagnoses were coded using the ICD10 system.^21^

### Patients, Regions, Confounders

The study cohort was selected by queries from the database. Patients aged ≥18 years who were discharged from the hospital between January 2012 and July 2015 with the principal ICD10 diagnosis I21, AMI, and had at least 3 months of follow-up before AMI were eligible for the analysis. Patients were assigned to the political districts of their residence. Because patients might have experienced more than one event in the observational period, we considered the chronologically first recorded diagnosis of AMI for each patient as the index event.

Potential confounders that could be associated with both the outcomes and the regional assignment included patients’ age, sex, and the year (2012–2015) of diagnosis of AMI and patients’ medical history as traceable through recorded diagnoses in billing sets and filled prescriptions. In particular, these were comorbidities (grouped in 22 ICD10 chapters), total length of hospitalization days, and co-medication (grouped in 96 second-level ATC codes), all collected from a window of 3 months before AMI. In addition, we also included secondary diagnoses recorded during hospitalization due to AMI as confounders. This resulted in 122 potential confounders. There were no missing data, and implausible data had been cleaned in the source data.

### Primary Outcome Measures

Patients were followed from the date of discharge from the hospital until the cessation of high-intensity statin therapy, 12 months of follow-up, administrative censoring, or until death or recurrent AMI that were considered as competing events. A patient was considered as compliant if high-intensity statin was continuously supplied over the period of 12 months after AMI. The continuous supply of the therapy was assessed by dividing the cumulative dosage dispensed by the days supplied. Cessation was identified if patients did not refill a new drug claim for over 30 days beyond the expected continuous therapy supply date. Furthermore, a tolerance of 2 more days was introduced in case of inpatient hospitalization as hospitals typically manage therapy during this period, and likewise, we considered that the therapy cessation could not have occurred during inpatient hospitalization.

The estimands of interest were the expected time in compliance lost within 12 months after AMI due to nonadherence or other competing events (death, recurrent AMI), and the expected time in compliance within 12 months after AMI. The expected times were compared between Austria’s political districts. In addition, for each political district, we calculated the centered estimates of the expected patients’ time in compliance lost as the difference between the regional estimates and the overall risk-adjusted estimate obtained by averaging the regional estimates of overall political districts. To quantify the uncertainties in our estimates by means of CIs, we used the bias-corrected bootstrap, resampling 250 times the original data set with replacement, each time maintaining the original cohort size. These CIs were Bonferroni-adjusted for multiple comparisons, controlling an overall confidence level of 95%.

### Approaches

The resulting estimates of our target parameters were obtained by using inverse probability of treatment weighting (IPTW), outcome regression, and doubly robust estimation. We obtained the propensity scores ${\hat{e}}_{z}\left(X\right)$ from a multinomial logistic regression model that included all the confounders. To avoid convergence issues because of a large number of region-specific probabilities requiring estimation, we applied the ridge penalty to the multinomial likelihood. The penalty strength was determined by optimizing the 10-fold cross-validated multinomial deviance. Pseudo-observations of RMTL due to nonadherence and competing events at τ=12 months were computed for each political district z separately. These pseudo-observations were then either combined with IPTW or used as a response variable in linear regression models, estimating region-specific intercepts and coefficients associated with the confounders to obtain risk-adjusted estimates. In both the propensity score model and the outcome models, we assumed a nonlinear functional form of the effect of age, modelled by natural splines with 4 degrees of freedom, and an interaction between age and sex. We computed doubly robust estimates by combining IPTW estimation with outcome regression. To compare results obtained by applying alternative estimands (Table 1), we also estimated the intensity of noncompliance up to 12 months by a multivariable Poisson regression model with weights to adjust for shorter follow-up due to competing events. These weights were defined as the proportion of follow-up time available for a patient within 12 months.

All analysis was performed in R,^22^ version 4.2.2, using packages glmnet,^23^ pseudo,^24^ and prodlim.^25^

## RESULTS

We identified (n=31,678) unique patients aged ≥18, residing in (*m*=116) political districts of Austria (a second-level division), who were eligible for the analysis. The median (25th and 75th percentile) age of patients was 69 (57, 79) years and 11,518 (36%) patients were female. In median (25th and 75th percentile) the political districts consisted of 257 (164, 363) patients but the range (9, 784) was large. The full list of confounders and the distribution of patients across provinces (a first-level division) is given in Supplemental Table 1 (Supplemental Digital Content 1, http://links.lww.com/MLR/C883).

The unadjusted mean time in compliance lost due to nonadherence was 6.3 months, corresponding to 20,853 noncompliant patients, and due to competing events was 1.2 months, corresponding to 3331 patients with competing events. Supplemental Figure 1 (Supplemental Digital Content 1, http://links.lww.com/MLR/C883) shows the cumulative incidence of noncompliance due to nonadherence and due to competing events of the total population with cumulative numbers of noncompliant patients. The unadjusted estimates across distinct political districts ranged from 3.7 to 9.1 months with respect to nonadherence and from 0 to 4.1 months with respect to competing events. Thus, the overall unadjusted mean time in compliance within 12 months was 4.5 months, ranging from 1.8 to 7.4 months across the political districts, or, equivalently, the mean proportion of time in compliance was 4.5/12×100=37.5% of the total 12 months covered by this PI.

**FIGURE 3:**
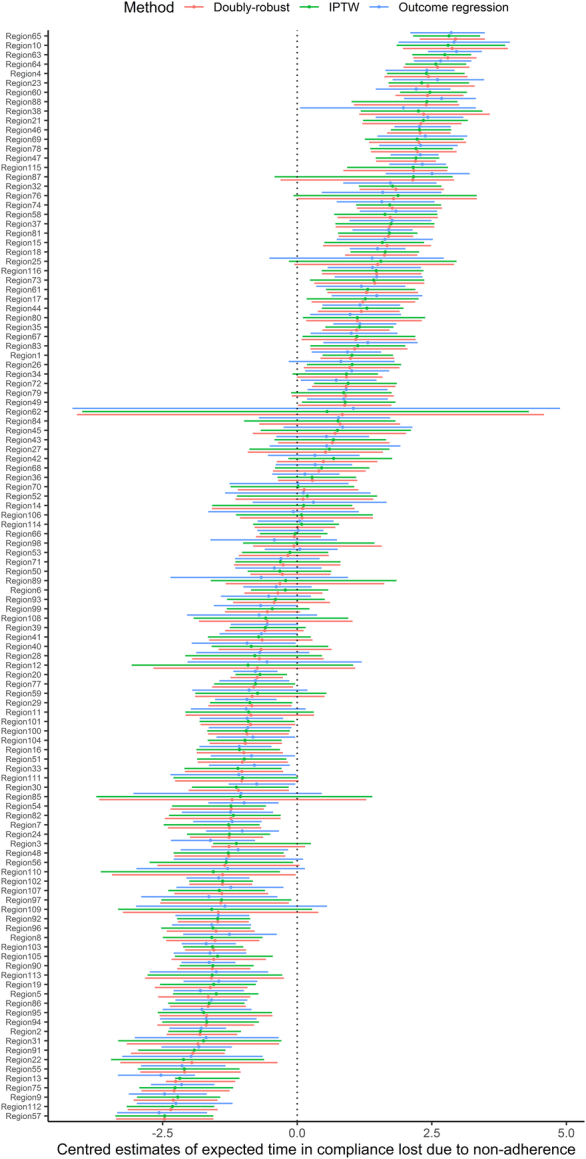
Comparison of expected compliance time lost due to nonadherence and associated multiplicity-adjusted 95% CIs between 116 political districts in Austria. IPTW indicates inverse probability of treatment weighting.

In Supplemental Figure 2 (Supplemental Digital Content 1, http://links.lww.com/MLR/C883), the distribution of propensity scores for each political district across the entire patient population, stratified by confounder set, is shown. Figure 3 compares the centered regional estimates of expected time in compliance lost due to nonadherence obtained by the 3 methods. Generally, there was a good agreement among the 3 approaches, with results disagreeing for a few political districts only. The interval estimates from the outcome regression approach were generally shorter than those obtained by the IPTW and the doubly robust estimators. This may indicate the lower efficiency of the latter 2 methods, or it may more accurately indicate the inherent uncertainty associated with the estimates. Comparing RMTL to the intensity of noncompliance across entities, there were some similarities but also deviations between the estimates (Supplemental Fig. 3, Supplemental Digital Content 1, http://links.lww.com/MLR/C883). In particular, when RMTL was still less than 12 months, the intensity of noncompliance was already saturated at 1, showing RMTLs higher sensitivity to partial compliance.

**FIGURE 4:**
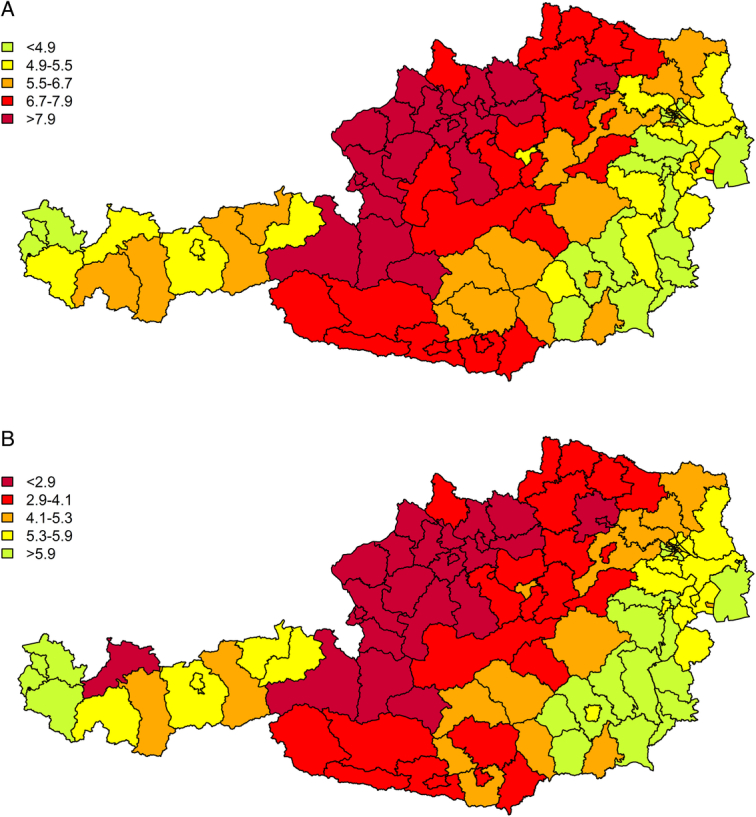
(A) Doubly robust estimates of adjusted restricted mean time lost due to nonadherence within 12 months in political districts of Austria. (B) Doubly robust estimates of adjusted restricted mean time in compliance.

Figure 4 and Supplemental Figure 4 (Supplemental Digital Content 1, http://links.lww.com/MLR/C883) show the doubly robust estimates of the parameters of interest and display substantial geographic variation in expected time in compliance (lost due to nonadherence) between 0 and 12 months. While no spatial smoothing was applied, Figure 4 shows a clearly visible spatial association of the estimates.

## DISCUSSION

We proposed RMST and RMTL as novel target estimands for PI. They offer an intuitive and attractive interpretation as expected time in compliance before time τ, or, alternatively, the fraction of the total time covered by the PI, and are well-suited for risk-adjusted entity comparisons in the presence of (measurable) confounding, censoring, and competing risks. They are easily communicable to the public, health policy makers, and other stakeholders, which is crucial for informed decision-making in public health. Their key advantage compared with alternative estimands lies in their ability to encapsulate compliance to treatment guidelines across a well-defined period rather than focussing on a single time horizon. Consequently, they can summarize the difference between the 2 entities when noncompliance initially diverges and later converges,^26^ giving more weight to compliance in the first months, which is probably more relevant for a patient than adherence close to time τ. Nevertheless, PI usually has a natural restriction time τ equal to the recommended duration of treatment. In the absence of a natural restriction time, some clinically relevant duration should be considered as restriction time (for the impact of data-driven choices of the restriction time, see Tian et al^27^), or the analysis could be extended using multiple restriction times.^28^

The proposed estimators are easily constructed, require no constant or proportional hazards or other distributional assumptions (contrary to Poisson regression that assumes a constant hazard) and are implementable via available software. Specifically, RMST and RMTL can be modelled using pseudo-observations, serving as a surrogate for the incompletely observed outcome. Consequently, formal causal comparisons can be obtained by applying standard methods for the completely observed outcome, for example, IPTW, outcome regression, and doubly robust estimation.^29^ A limitation is that there must be enough participants at risk at the appearance of each case to prevent imprecise estimates of pseudo-observations.^30^ Moreover, the interpretability and comparability of these standardized estimates of compliance across entities depend on the validity of causal assumptions which are common for addressing causal questions.^31^ Some assumptions, however, are untestable. For example, “no unmeasured confounding” assumes that the potential outcomes are independent of the entity given the considered confounders. Even though the double robustness property offers some protection against bias from unmeasured confounding,^32^ it does not eliminate the problem. Therefore, incorporating numerous potential confounders may enhance the credibility of this assumption.^7^ We included confounders that could be associated with both the outcomes and the regional assignment of patients, considering their demographic characteristics and medical history, collected from a window of 3 months before AMI. Extending this window to at least 12 months could be advantageous to prevent overlooking relevant comorbidities.

Demonstrating the application of RMST and RMTL for assessing PI, we estimated the expected time in compliance (lost) to high-power statin therapy during the first 12 months after AMI in Austria’s political districts. While quality indicators are typically used to compare the quality of care between health care providers, our cross-sectoral results show the combined regional differences in providers’ performances, accessibility to health services, and patient behavior. In our analysis, noncompliance was defined as noncoverage with high-power statins for at least 30 days and was a nonrevertible state. Future work may concentrate on situations where patients have periods of noncompliance followed by compliance. This can be addressed by different estimands such as the medication possession ratio, or by varying the length of the tolerance window. We also narrowed this tolerance window from 30 to 7 days, but differences in the estimates were negligible. In practice, one should explore various time windows to assess the sensitivity of the resulting estimates to different cut-off points.^28^

Our propensity and outcome models included all available potential confounders, which may contain information that can significantly enhance results' accuracy and interpretation. With a substantial number of regional probabilities to be estimated, issues with convergence may arise in fitting multinomial regression models. Rather than covariate preselection based on domain knowledge or observed associations, shrinkage has been shown to be the preferred approach.^33^ Thus, we used ridge regression to regularize the estimates of the multinomial propensity scores. While our analysis focused on estimating the overall risk-adjusted estimates for each residential unit, often, the interest lies in identifying risk factors of nonadherence. Outcome model estimation based on pseudo-observations allows for simultaneous inference in general parametric models, and the uncertainty of coefficient estimates may be quantified by applying the sandwich estimator.^34^

Our results, employing RMST and RMTL, exhibited regional differences across Austria’s political districts of residence but also showed plausible spatial similarities. One could involve spatial smoothing because neighboring regions tended to behave similarly with respect to compliance. The exceptions are perhaps small urban-like districts surrounded by rural districts. These districts sometimes showed disparate compliance patterns but also exhibited large uncertainty, which was reflected in wide confidence intervals.

In summary, RMST is an attractive novel approach to compare the quality of health services across entities, in particular, if the evaluation of long-term treatment is the focus of analysis. Software to estimate RMST using pseudo-observations is available within statistical software packages.

## Supplementary Material
