# Supplementary material for: Cross-Sectoral Comparisons of Process Quality Indicators of Health Care Across Residential Regions Using Restricted Mean Survival Time
Source: Med Care. 2024 Oct 11;62(11):748–56. doi: 10.1097/MLR.0000000000002057 (PMC11462882; doi:10.1097/MLR.0000000000002057)
Supplement: Supplementary file 1 [file mlr-62-748-s001.pdf]

Cross-sectoral comparisons of procedural quality indicators of health care across residential regions using restricted mean survival time

Supplement

Contents

S1. Pseudo-observations 2

S2. Causally modeling RMST for risk adjusted comparisons of entities 4

Supplemental Table 1 6

Supplemental Figure 1 9

Supplemental Figure 2 10

Supplemental Figure 3 11

Supplemental Figure 4 12

References 13

S1. Pseudo-observations

In observational studies one would like to include into analysis patients' baseline covariates X that influence both the outcomes and patients' assignment to centre Z to perform risk adjustment and to identify risk factors related to non-adherence. Apart from inconvenient indirect approaches that employ classical time-to-event methods to relate RMST to the baseline covariates, e.g., cause-specific hazard Cox models or a Fine–Gray proportional subdistribution hazards model [1, 2], there are several existing alternatives that model the RMST as a function of baseline covariates directly, e.g., by pseudo-observations [3], by inverse probability of censoring weighting [4], or via generalized estimating equations [5]. In our application that focuses on a single time point τ we will demonstrate how one can combine causal inference methods with pseudo-observations to model the time to event of interest, subject to competing events, without censoring issue [6]. The pseudo-observations can be easily calculated via available software [7, 8].

A pseudo-observation for each patient i $(i = 1, \dots, n)$ in the sample of size n is constructed based on jack-knife statistic

$$\hat{\theta}_i = n\hat{\theta} - (n-1)\hat{\theta}^{-i},$$

where $\hat{\theta}$ is a consistent estimator for the parameter of interest $\theta = E(f(Y))$ with f denoting some function of the survival time Y and $\hat{\theta}^{-i}$ is the estimator applied to the sample, eliminating subject i. Intuitively, a pseudo-observation can be interpreted as the contribution of subject i to the target estimate on the sample of size n [9]. Thus, the i-th pseudo-observation for the cumulative incidence function is

$$\hat{\theta}_{i,j}(t) = n\hat{F}_j(t) - (n-1)\hat{F}_{-i,j}(t),$$

where $\hat{F}_j(t)$ is the Aalen-Johansen estimator for the cumulative incidence function of cause j. Because $\hat{\theta}_{i,j}(t)$ is a stepwise function, changing at event times only, the i-th pseudo-observation for the RMTL to cause j can be obtained as

$$\hat{\mu}_{i,j} = \int_0^{\tau} \hat{\theta}_{i,j}(t)dt = (\tau - t_\tau)\hat{\theta}_{i,j}(t_\tau) + \sum_{t_k \le t_\tau} (t_k - t_{k-1})\, \hat{\theta}_{i,j}(t_{k-1}),$$

where $t_k, k = 1, \dots, K,$ are K unique ordered event times, $t_0 = 0$ and $t_\tau \le \tau$ is the last event time before τ [10].

Throughout this paper we will assume that censoring is independent of event times and covariates (X, Z). For such situations it has been shown that $E(\hat{\theta}_i | X_i, Z_i = z) \approx E(f(Y_i) | X_i, Z_i = z)$ [6, 11]. Thus, the idea is to replace the incompletely observed random variable $f(Y_i)$ by $\hat{\mu}_{i,j}$ to perform direct confounding adjustment by modelling $\hat{\mu}_{i,j}$ using a generalized linear model or to combine $\hat{\mu}_{i,j}$ with propensity score weighting [6]. Alternatively, one may consider to use a doubly-robust estimator that combines inverse probability of treatment weighting (IPTW) and the regression model-based adjustment approach to achieve robustness to model misspecification.

S2. Causally modeling RMST for risk adjusted comparisons of entities

Consider the set of potential time to event outcomes $Y^z, Z = 1, \dots, m,$ had all patients been assigned to entity $Z = z$. The goal of our analysis is to estimate the causal RMTL to cause j before τ, setting the entity to z, as a parameter of the distribution of the potential outcomes Y^z [10].

The following assumptions are required to interpret estimates of Y^z as causal as only one of the potential outcomes is actually realized [12]. First, *consistency* relates the observed and potential outcomes by $Y_i = Y_i^{z_i}$. Second, under *positivity* every patient should have a probability greater than 0 and smaller than 1 for being assigned to entity z. Third, *no unmeasured confounders* requires that all relevant confounders are contained in the data. Finally, *stable treatment unit assumption* assumes that the entity to which a patient was assigned to does not interfere with the potential outcomes for other patients and that there is no variation within entities.

One standard approach for causal inference is based on IPTW. The unknown propensity score, $e_z(X_i) = P(Z_i = z|X_i)$, defined as the vector of probabilities to belong to each entity z based on patient's baseline covariates X_i, can be parametrized as $e_z(X_i; \gamma)$, where γ can be estimated by maximizing the multinomial likelihood. A re-weighted data set in which allocation to entity z is independent of measured covariates is then created by using the weights $\widehat{w}_z(X) = 1/\hat{e}_z(X)$. The weighted average pseudo-observations in each entity

$$\hat{\mu}_{j,z}^{IPTW} = \frac{\sum_{i=1}^{n} I\{Z_i = z\}\hat{\mu}_{i,j}\widehat{w}_z(X_i)}{\sum_{i=1}^{n} I\{Z_i = z\}\widehat{w}_z(X_i)},$$

can be used as an estimator of the causal RMTL to cause j before time τ.

Alternatively, one can perform direct confounding adjustment using pseudo-observations in the outcome regression model of form

$$\mu_{i,j} = \alpha_z + \beta^T X_i,$$

where α_z is the entity-specific effect. The standard errors can be computed by the sandwich estimator [13]. Thus, once pseudo-observations have been computed, the strength of risk factors of non-adherence and their standard errors can be estimated using standard statistical software for generalized estimating equations, though the standard error estimates may be slightly conservative [14]. The causal RMTL to event type j before time τ can be then obtained via g-computation

$$\hat{\mu}_{j,z}^{G} = \frac{1}{n}\sum_{i=1}^{n} E\left(\hat{\mu}_{i,j} \middle| X_i, Z_i = z\right),$$

averaging the conditional expectation of the transformed potential survival outcome given covariates.

The IPTW estimator and the G-formula estimator are unbiased estimators of $E(Y^z)$ only if statistical models for the propensity score and outcome regression, respectively, are correctly specified. As proposed in Wang [2] for $m = 2$ and extended in Zeng [15] for $m \geq 2$, the IPTW estimator can be further augmented by the outcome regression model to obtain the doubly-robust estimator

$$\hat{\mu}_{j,z}^{DR} = \frac{1}{n}\sum_{i=1}^{n} E\left(\hat{\mu}_{i,j} \middle| X_i, Z_i = z\right) + \frac{\sum_{i=1}^{n} I\{Z_i = z\}(\hat{\mu}_{i,j} - E(\hat{\mu}_{i,j}|X_i, Z_i = z))\widehat{w}_z(X_i)}{\sum_{i=1}^{n} I\{Z_i = z\}\widehat{w}_z(X_i)}$$

that remains consistent as long as one of both models is correctly specified [16, 17]. That is the case if either regression accounts for confounders in a way that reflects their true relationship to entities and outcomes. Using doubly-robust estimates is appealing in practice because the true structure of the regression is usually unknown, and the doubly-robust nature allows for misspecification of one of the two models [18].

Once the estimates of RMTL due to each event type j before time τ are obtained, the causal RMTL before time τ can be expressed as the average of the following terms

$$E(Y^z \wedge \tau) = \tau - \sum_{j=1}^{J} \int_0^{\tau} \hat{F}_j(t|X, Z = z)dt = \tau - \sum_{j=1}^{J} \hat{\mu}_{j,z}\,.$$

The uncertainty of those estimates can be assessed by the non-parametric bootstrap. Here, the observations in the data set are resampled with replacement, and for each resample the propensity score model is re-estimated and pseudo-observations reconstructed to estimate RMTL [6, 19]. Alternatively, one may resort to an asymptotic variance expression proposed by Zeng [15] which can provide computationally efficient inference without resampling.

Supplemental Table 1

Characteristics of the $n = 31{,}678$ patients		
Median age, years (25th, 75th percentile)		69 (57, 79)
Female - no. (%)		11518 (36.4)
Province - no. (%)		
Burgenland		1139 (3.6)
Carinthia		2496 (7.9)
Lower Austria		6337 (20.0)
Upper Austria		5297 (16.7)
Salzburg		1627 (5.1)
Styria		4478 (14.1)
Tyrol		2703 (8.5)
Vorarlberg		1373 (4.3)
Vienna		6228 (19.7)
Year of AMI - no. (%)		
	2012	8778 (27.7)
	2013	8922 (28.2)
	2014	9121 (28.8)
	2015	4857 (15.3)
Patients hospitalized before AMI - no. (%)		2989 (9.4)
Median length of hospitalization before AMI conditional there were hospitalizations, days (25th, 75th percentile)		6 (3-11)
ICD-10 chapters		
I Certain infectious and parasitic diseases - no. (%)		694 (2.2)
II Neoplasms - no. (%)		176 (0.6)
III Diseases of the blood and blood-forming organs and certain disorders involving the immune mechanism - no. (%)		951 (3.0)
IV Endocrine, nutritional and metabolic diseases - no. (%)		14306 (45.2)
V Mental and behavioural disorders - no. (%)		5250 (16.6)
VI Diseases of the nervous system - no. (%)		1577 (5.0)
VII Diseases of the eye and adnexa - no. (%)		690 (2.2)
VIII Diseases of the ear and mastoid process - no. (%)		242 (0.8)
IX Diseases of the circulatory system - no. (%)		24348 (76.9)
X Diseases of the respiratory system - no. (%)		3871 (12.2)
XI Diseases of the digestive system - no. (%)		2551 (8.1)
XII Diseases of the skin and subcutaneous tissue - no. (%)		439 (1.4)
XIII Diseases of the musculoskeletal system and connective tissue - no. (%)		2286 (7.2)
XIV Diseases of the genitourinary system - no. (%)		4130 (13.0)
XVII Congenital malformations, deformations and chromosomal abnormalities - no. (%)		164 (0.5)
XVIII Symptoms, signs and abnormal clinical and laboratory findings, not elsewhere classified - no. (%)		3357 (10.6)
XIX Injury, poisoning and certain other consequences of external causes - no. (%)		1407 (4.4)
XX External causes of morbidity and mortality - no. (%)		871 (2.7)

XXI Factors influencing health status and contact with health services - no. (%)	1430 (4.5)
ATC 2nd level	
A01 stomatological preparations - no. (%)	225 (0.7)
A02 drugs for acid related disorders - no. (%)	9960 (31.4)
A03 drugs for functional gastrointestinal disorders - no. (%)	815 (2.6)
A04 antiemetics and antinauseants - no. (%)	17 (0.1)
A05 bile and liver therapy - no. (%)	314 (1.0)
A06 drugs for constipation - no. (%)	1569 (5.0)
A07 antidiarrheals, intestinal antiinflammatory/antiinfective agents - no. (%)	828 (2.6)
A09 digestives, incl. enzymes - no. (%)	178 (0.6)
A10 drugs used in diabetes- no. (%)	5010 (15.8)
A11 vitamins - no. (%)	1534 (4.8)
A12 mineral supplements - no. (%)	2430 (7.7)
A14 anabolic agents for systemic use - no. (%)	14 (0.0)
A16 other alimentary tract and metabolism products - no. (%)	33 (0.1)
B01 antithrombotic agents - no. (%)	5358 (16.9)
B02 antihemorrhagics - no. (%)	23 (0.1)
B03 antianemic preparations - no. (%)	808 (2.6)
B05 blood substitutes and perfusion solutions - no. (%)	545 (1.7)
C01 cardiac therapy - no. (%)	3514 (11.1)
C02 antihypertensives - no. (%)	1743 (5.5)
C03 diuretics - no. (%)	2834 (8.9)
C04 peripheral vasodilators - no. (%)	708 (2.2)
C05 vasoprotectives - no. (%)	1738 (5.5)
C07 beta blocking agents - no. (%)	6759 (21.3)
C08 calcium channel blockers - no. (%)	4083 (12.9)
C09 agents acting on the renin-angiotensin system - no. (%)	12317 (38.9)
C10 lipid modifying agents - no. (%)	7392 (23.3)
D01 antifungals for dermatological use - no. (%)	582 (1.8)
D02 emollients and protectives - no. (%)	56 (0.2)
D03 preparations for treatment of wounds and ulcers - no. (%)	196 (0.6)
D04 antipruritics, incl. antihistamines, anesthetics, etc. - no. (%)	40 (0.1)
D05 antipsoriatics - no. (%)	164 (0.5)
D06 antibiotics and chemotherapeutics for dermatological use - no. (%)	368 (1.2)
D07 corticosteroids, dermatological preparations - no. (%)	994 (3.1)
D08 antiseptics and disinfectants - no. (%)	366 (1.2)
D10 anti-acne preparations - no. (%)	63 (0.2)
D11 other dermatological preparations - no. (%)	371 (1.2)
G01 gynecological antiinfectives and antiseptics - no. (%)	41 (0.1)
G02 other gynecologicals - no. (%)	42 (0.1)
G03 sex hormones and modulators of the genital system - no. (%)	464 (1.5)
G04 urologicals - no. (%)	2572 (8.1)
H01 pituitary and hypothalamic hormones and analogues - no. (%)	10 (0.0)
H02 corticosteroids for systemic use - no. (%)	1388 (4.4)
H03 thyroid therapy - no. (%)	1956 (6.2)
H05 calcium homeostasis - no. (%)	53 (0.2)
J01 antibacterials for systemic use - no. (%)	4767 (15.0)

J02 antimycotics for systemic use - no. (%)	78 (0.2)
J04 antimycobacterials - no. (%)	17 (0.1)
J05 antivirals for systemic use - no. (%)	207 (0.7)
L01 antineoplastic agents - no. (%)	40 (0.1)
L02 endocrine therapy - no. (%)	218 (0.7)
L03 immunostimulants - no. (%)	89 (0.3)
L04 immunosuppressants - no. (%)	388 (1.2)
M01 antiinflammatory and antirheumatic products - no. (%)	6411 (20.2)
M02 topical products for joint and muscular pain - no. (%)	1538 (4.9)
M03 muscle relaxants - no. (%)	764 (2.4)
M04 antigout preparations - no. (%)	1071 (3.4)
M05 drugs for treatment of bone diseases - no. (%)	1097 (3.5)
N01 anesthetics - no. (%)	281 (0.9)
N02 analgesics - no. (%)	3649 (11.5)
N03 antiepileptics - no. (%)	1276 (4.0)
N04 anti-parkinson drugs - no. (%)	788 (2.5)
N05 psycholeptics - no. (%)	2879 (9.1)
N06 psychoanaleptics - no. (%)	5810 (18.3)
N07 other nervous system drugs - no. (%)	397 (1.3)
P01 antiprotozoals - no. (%)	86 (0.3)
R01 nasal preparations - no. (%)	540 (1.7)
R02 throat preparations - no. (%)	226 (0.7)
R03 drugs for obstructive airway diseases - no. (%)	3315 (10.5)
R05 cough and cold preparations - no. (%)	1219 (3.8)
R06 antihistamines for systemic use - no. (%)	911 (2.9)
S01 ophthalmologicals - no. (%)	2064 (6.5)
S02 otologicals - no. (%)	52 (0.2)
S03 ophthalmological and otological preparations - no. (%)	126 (0.4)
V03 all other therapeutic products - no. (%)	123 (0.4)

Supplemental Table 1. Characteristics of patients at AMI. ICD-10 chapters and ATC codes refer to hospitalizations and prescriptions filled in a look-back window of 90 days before the AMI index event and secondary diagnoses recorded during hospitalization due to AMI. Characteristics with absolute frequency less than or equal to 10 patients are not shown.

Supplemental Figure 1

Supplemental Figure 1: Cumulative incidence of non-adherence due to non-compliance (solid line) and due to competing events (dotted line) of the total population with cumulative numbers of non-adhering patients.

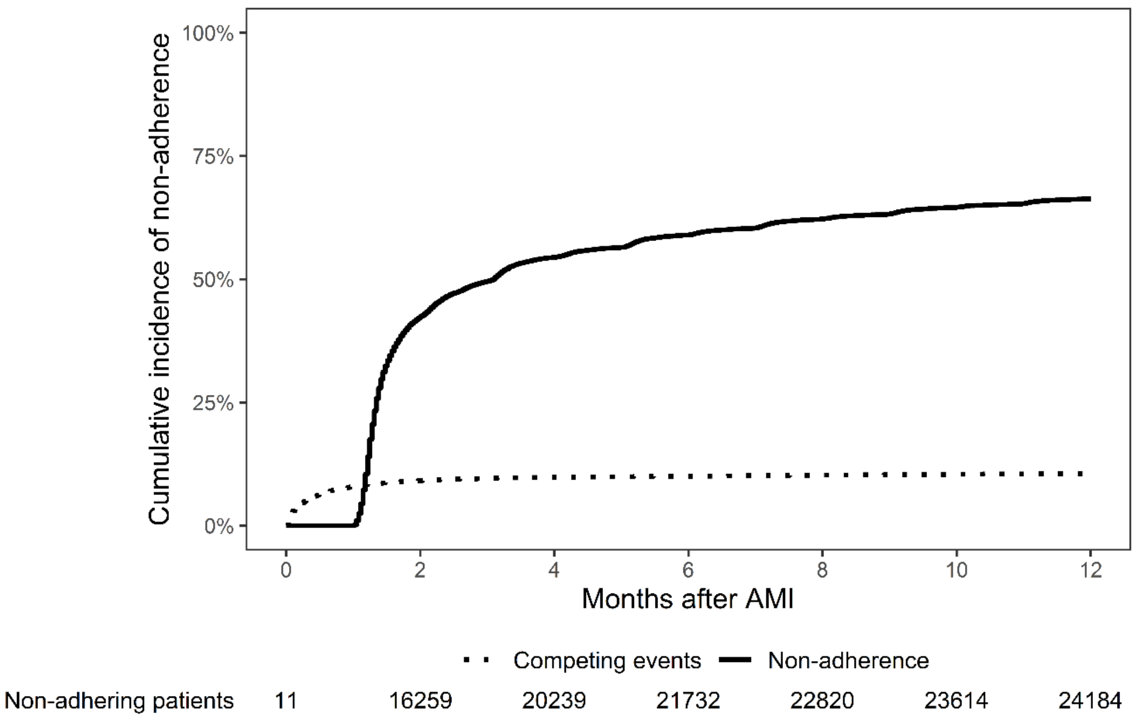

Supplemental Figure 2

Supplemental Figure 2: Distribution (density estimates) of propensity scores in 116 political districts of Austria based on multinomial logistic regression models including as covariates demographics, hospitalizations and filled prescriptions in time windows of 180 days before AMI. The shown propensity scores are the probabilities of the assigned district.

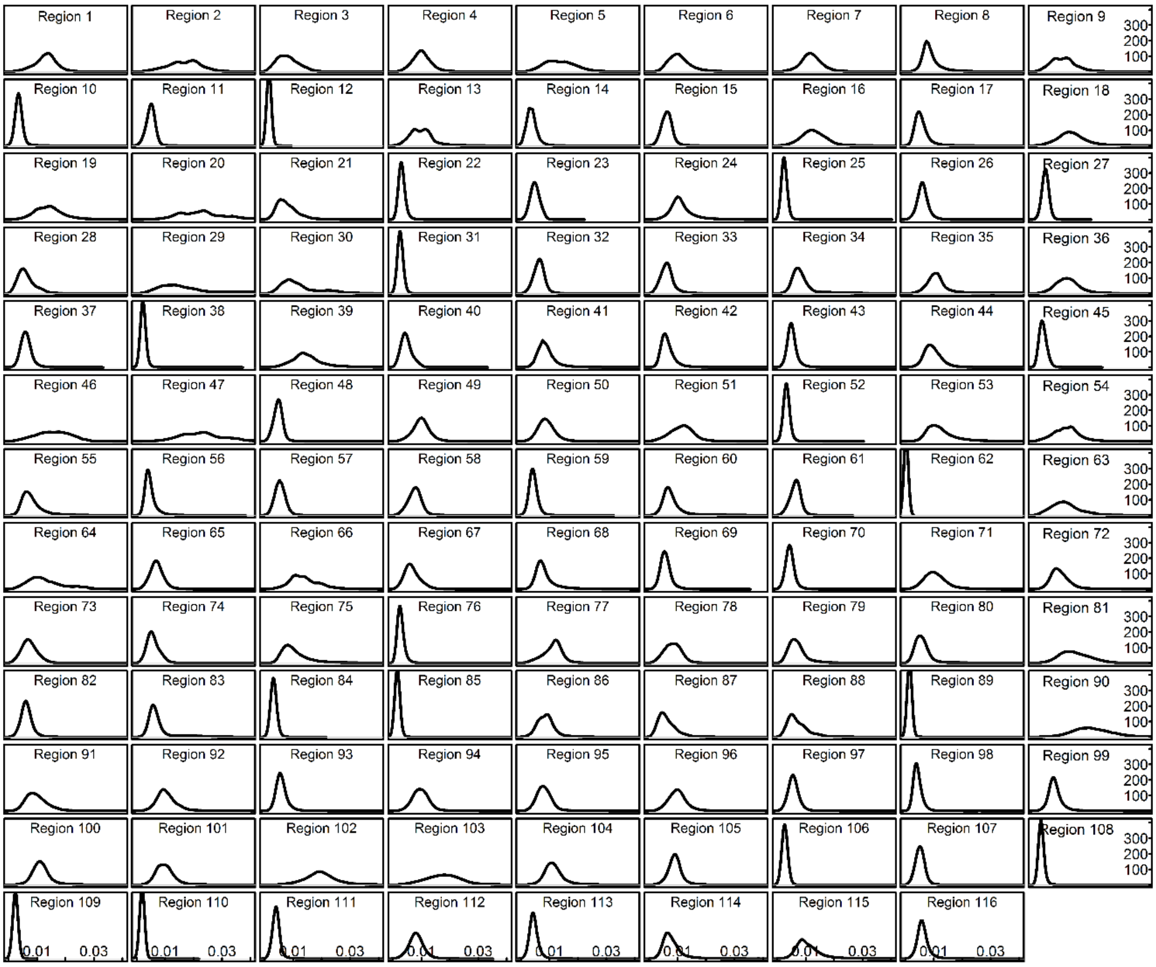

Supplemental Figure 3

Supplemental Figure 3: Estimates of expected time in compliance lost due to non-adherence obtained by outcome model approach (x-axis) and intensity of non-compliance (y-axis) across entities.

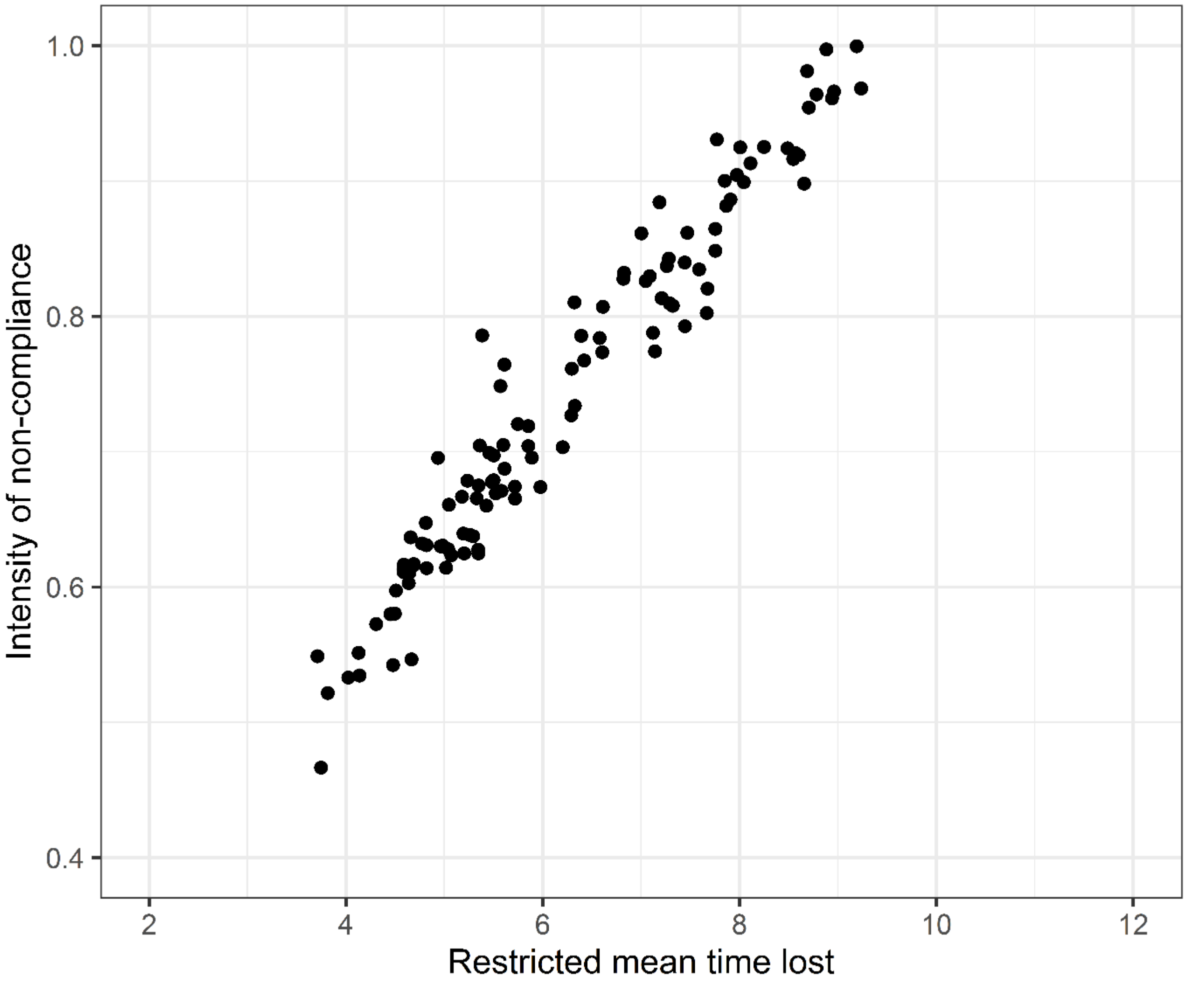

Supplemental Figure 4

Supplemental Figure 3: Doubly-robust estimates of adjusted restricted mean time in compliance within 12 months from AMI (RMST), restricted mean time lost due to competing events (RMTL competing event) and restricted mean time lost due to non-adherence in 116 political districts in Austria.

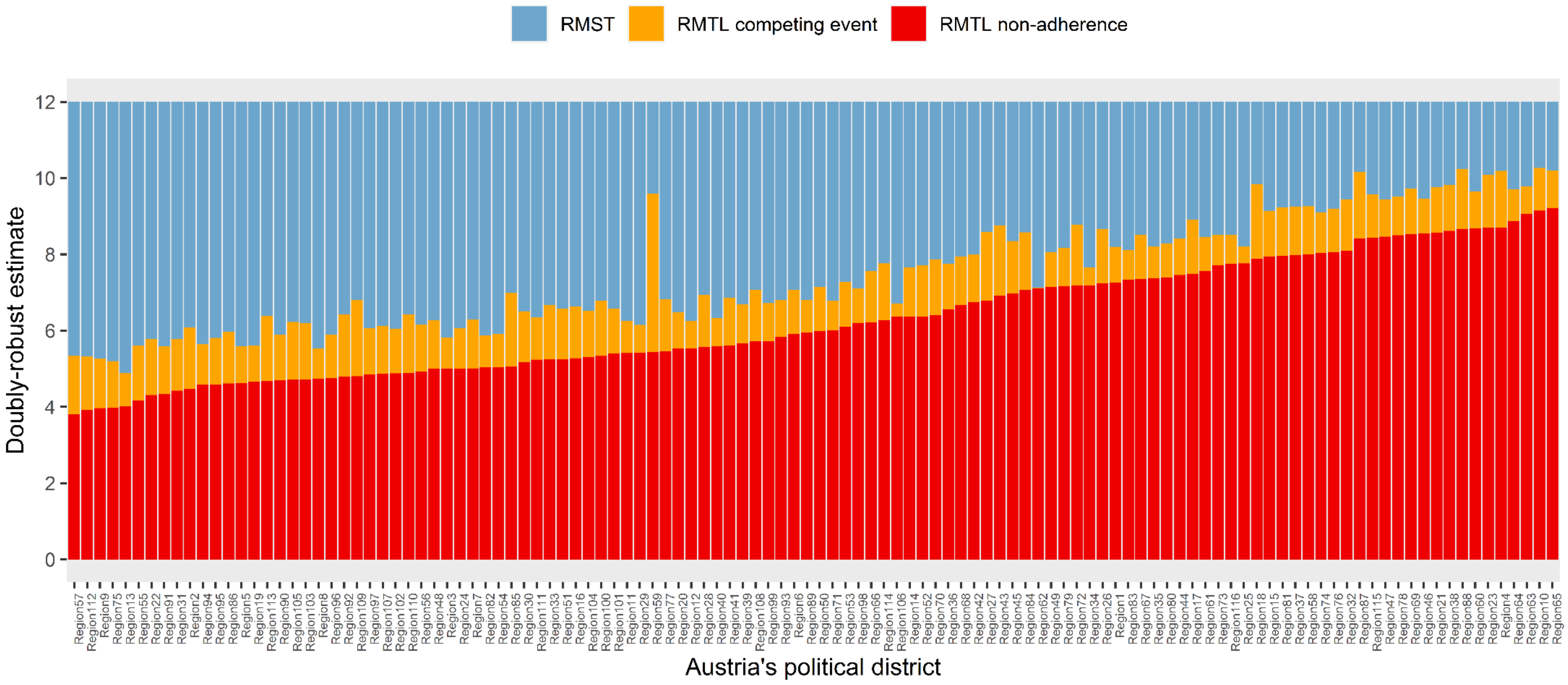

References

1. Rong R, Ning J, Zhu H. Regression modeling of restricted mean survival time for left-truncated right-censored data. Stat Med. 2022 Jul 20;41(16):3003-3021. doi: 10.1002/sim.9399.
2. Wang J. A simple, doubly robust, efficient estimator for survival functions using pseudo observations. Pharm Stat. 2018 Feb;17(1):38-48. doi: 10.1002/pst.1834
3. Andersen PK, Hansen MG, Klein JP. Regression analysis of restricted mean survival time based on pseudo-observations. Lifetime Data Anal. 2004 Dec;10(4):335-50. doi: 10.1007/s10985-004-4771-0
4. Tian L, Zhao L, Wei LJ. Predicting the restricted mean event time with the subject's baseline covariates in survival analysis. Biostatistics 2014 15(2): 222–233. doi: 10.1093/biostatistics/kxt050
5. Wang X, Schaubel DE. Modeling restricted mean survival time under general censoring mechanisms. Lifetime Data Anal. 2018 Jan;24(1):176-199. doi: 10.1007/s10985-017-9391-6
6. Andersen PK, Syriopoulou E, Parner ET. Causal inference in survival analysis using pseudo-observations. Stat Med. 2017 Jul 30;36(17):2669-2681. doi: 10.1002/sim.7297.
7. Pohar Perme M, Gerster M, Rodrigues K. pseudo: Computes Pseudo-Observations for Modeling. R package Version 1.4.3, July 30, 2017. Available at: https://cran.r-project.org/package=pseudo. Accessed October 31, 2023
8. Gerds TA. prodlim: Product-Limit Estimation for Censored Event History Analysis. R package Version 2023.08.28. August 28, 2023. Available at: https://cran.r-project.org/package=prodlim. Accessed October 31, 2023

9. Andersen PK, Perme MP. Pseudo-observations in survival analysis. Stat Methods Med Res. 2010 Feb;19(1):71-99. doi: 10.1177/0962280209105020
10. Lin J, Trinquart L. Doubly-robust estimator of the difference in restricted mean times lost with competing risks data. Stat Methods Med Res. 2022 Oct;31(10):1881-1903. doi: 10.1177/09622802221102625.
11. Graw F, Gerds TA, Schumacher M. On pseudo-values for regression analysis in competing risks models. Lifetime Data Anal. 2009 Jun;15(2):241-55. doi: 10.1007/s10985-008-9107-z.
12. Goetghebeur E, le Cessie S, De Stavola B, et al, on behalf of the topic group Causal Inference (TG7) of the STRATOS initiative. Formulating causal questions and principled statistical answers. Stat Med. 2020 Dec 30;39(30):4922-4948. doi: 10.1002/sim.8741
13. Andersen PK, Klein JP, Rosthøj S. Generalised linear models for correlated pseudo-observations, with applications to multi-state models, Biometrika 2003 90(1): 15–27. doi: 10.1093/biomet/90.1.15
14. Ambrogi F, Iacobelli S, Andersen PK. Analyzing differences between restricted mean survival time curves using pseudo-values. BMC Med Res Methodol. 2022 Mar 18;22(1):71. doi: 10.1186/s12874-022-01559-z
15. Zeng S, Li F, Hu L, Li F. Propensity Score Weighting Analysis of Survival Outcomes Using Pseudo-observations (Version 3). arXiv 2021 2103.00605. doi: 10.48550/ARXIV.2103.00605
16. Varewyck M, Goetghebeur E, Eriksson M, et al. On shrinkage and model extrapolation in the evaluation of clinical center performance. Biostatistics. 2014 Oct;15(4):651-64. doi: 10.1093/biostatistics/kxu019.

17. Su CL, Platt RW, Plante JF. Causal inference for recurrent event data using pseudo-observations. Biostatistics. 2022 Jan 13;23(1):189-206. doi: 10.1093/biostatistics/kxaa020
18. Spertus JV, T Normand SL, Wolf R, et al. Assessing Hospital Performance After Percutaneous Coronary Intervention Using Big Data. Circ Cardiovasc Qual Outcomes. 2016 Nov;9(6):659-669. doi: 10.1161/CIRCOUTCOMES.116.002826
19. Austin PC. Variance estimation when using inverse probability of treatment weighting (IPTW) with survival analysis. Stat Med. 2016 Dec 30;35(30):5642-5655. doi: 10.1002/sim.7084
